# Preparation, Characterization, and In Vivo Evaluation of an Oral Multiple Nanoemulsive System for Co-Delivery of Pemetrexed and Quercetin

**DOI:** 10.3390/pharmaceutics10030158

**Published:** 2018-09-12

**Authors:** Rudra Pangeni, Vijay Kumar Panthi, In-Soo Yoon, Jin Woo Park

**Affiliations:** 1 Department of Pharmacy, College of Pharmacy and Natural Medicine Research Institute, Mokpo National University, Muan-gun, Jeonnam 58554, Korea; capriconpangeni@gmail.com (R.P.); nepalivijay7@gmail.com (V.K.P.); 2 Department of Manufacturing Pharmacy, College of Pharmacy, Pusan National University, Geumjeong-gu, Busan 46241, Korea

**Keywords:** pemetrexed, quercetin, multiple nanoemulsion, permeability, oral bioavailability, oral anticancer therapy

## Abstract

Co-administration of conventional and natural chemotherapeutics offers synergistic anticancer efficacy while minimizing adverse effects. In this study, an oral co-delivery system for pemetrexed (PMX) and quercetin (QCN) was designed based on water-in-oil-in-water nanoemulsion (NE), which is highly absorbable because it enhances the intestinal membrane permeability of PMX and aqueous solubility of QCN. To create this system, an ion-pairing complex of PMX with *N*^α^-deoxycholyl-l-lysyl-methylester (DCK) was formed and further incorporated with QCN into the NE, yielding PMX/DCK-QCN-NE. The results revealed synergistic inhibitory effects on human lung carcinoma (A549) cell proliferation and migration after combined treatment with PMX/DCK and QCN. The intestinal membrane permeability and cellular uptake of PMX/DCK and QCN from the NE were significantly improved via facilitated transport of PMX by the interaction of DCK with bile acid transporters, as well as NE formulation-mediated alterations in the membrane structure and fluidity, which resulted in 4.51- and 23.9-fold greater oral bioavailability of PMX and QCN, respectively, than each free drug. Tumor growth in A549 cell-bearing mice was also maximally suppressed by 62.7% after daily oral administration of PMX/DCK-QCN-NE compared with controls. Thus, PMX/DCK-QCN-NE is a promising oral nanocarrier of PMX and QCN for synergistic anticancer efficacy and long-term chemotherapy.

## 1. Introduction

The development of chemoresistance, incidence of unintended and painful adverse effects, and recurrence of secondary tumors are major drawbacks of chemotherapy [1,2]. To address these limitations, researchers have recently recommended the co-administration of anticancer drugs with natural chemotherapeutic agents, such as resveratrol, curcumin, and tetrandrine, to obtain synergistic anticancer efficacy while minimizing undesirable toxicity. The combined administration of these natural chemotherapeutic agents with conventional anticancer drugs showed increased antitumor activities by suppression of cancer cell proliferation, induction of apoptosis, and inhibition of cell survival pathways [3,4]. In addition, natural chemotherapeutic agents have been shown to inhibit the expression of P-glycoprotein (P-gp), which rapidly transports anticancer drugs out of cancer cells; they have also been used as sensitizing agents for the treatment of cancer by modulating multiple signaling pathways via inactivation of multiple drug resistance-related mRNAs [5,6,7].

Treatment of cancer cells by co-administration of drugs with different mechanisms of action has been proposed to attain synergistic anticancer effects and to minimize the doses of drugs required [8]. Recently, various novel formulations have been designed for the oral delivery of anticancer drugs to improve patients’ quality of life, reduce intravenous (IV) administration-related complications, reduce costs, increase suitability for prolonged treatment, and provide metronomic therapy [9]. Such oral metronomic therapy leads to sustained and low plasma concentrations of drugs targeting endothelial cells in the growing tumor vasculature, inhibiting angiogenesis and stimulating the immune system [10]. Thus, oral administration of anticancer drugs appears to be the most promising strategy for combination chemotherapy for long-term treatment, although problems remain with regard to oral bioavailability [11]. Furthermore, oral co-delivery is limited by the physicochemical properties of the drugs and physiological barriers, such as pre-systemic metabolism, gastrointestinal (GI) instability, low aqueous solubility, poor intestinal membrane permeability, and high levels of P-gp efflux [12,13]. Therefore, a great deal of research has been conducted to design drug delivery systems, such as liposomes and nanoparticles, for administration of two or more drug molecules in a single carrier [14,15,16].

Pemetrexed (*N*-[4-[2-(2-amino-3,4-dihydro-4-oxo-7H-pyrrolo[2,3-d]pyrimidin-5-yl)-ethyl]-benzoyl]-l-glutamic acid; PMX) is a US Food and Drug Administration-approved multi-targeted antifolate cytotoxic agent, clinically used in patients with several stages of non-squamous non-small cell lung cancer. Moreover, PMX shows enormous potential when used in combination with other targeted or immune therapies due to its good tolerability and low toxicity compared with other chemotherapeutic agents [17,18]. However, PMX is currently available only in IV injection form due to its low oral bioavailability caused by its low intestinal membrane permeability.

Quercetin (2-[3,4-dihydroxyphenyl]-3,5,7-trihydroxy-4H-chromen-4-one; QCN) is one of the most abundant natural flavonoids, present in numerous fruits and vegetables. It exhibits a wide range of physiological activities, such as anti-inflammatory and inhibitory effects on different human cancer cell lines [19,20]. In addition, the role of QCN as an anticancer agent is attributed to its potential to suppress the activity of cyclin-dependent kinases, antioxidative potential, induction of apoptosis by direct interaction with DNA, inhibition of enzymes that trigger carcinogenesis, alteration of signal transduction pathways, interactions with receptors and/or proteins, and inhibition of cell survival pathways [4,21,22]. Therefore, several studies have noted synergistic activity of QCN as a potential anticancer drug; with combined administration, it enhances the toxicity of platinum drugs to lung, colorectal, and ovarian cancer cells [23,24,25].

However, the physicochemical characteristics of QCN differ from those of PMX, including poor intestinal membrane permeability and low solubility in GI fluid, leading to poor oral bioavailability. To address these problems, various formulation strategies, such as synthesis of co-crystals, preparation of nanoemulsifying and self-emulsifying delivery systems, and creation of polymeric micelles, have been proposed to improve the physiochemical properties of QCN, thereby enhancing its oral bioavailability [26,27,28,29]. In addition, co-delivery of drugs with different properties requires the successful development of a single carrier or formulation that can incorporate both drugs. Among formulations, water-in-oil-in-water (w/o/w) multiple nanoemulsion (NE) can encapsulate hydrophilic and hydrophobic drugs simultaneously in the inner aqueous phase and outer oil/surfactant-co-surfactant phase, respectively. These multiple emulsions can also increase the solubility of poorly soluble drugs and protect the drug molecules from light, oxygen, and ions compared with normal emulsions [30]. Wang et al. (2011) prepared nanoparticles by the double emulsion method to incorporate hydrophilic doxorubicin and hydrophobic paclitaxel. The prepared nanoparticles showed well-controlled size distribution with high drug loading efficacy. Furthermore, the nanocarrier system for two anticancer drugs as a single formulation demonstrated enhanced cellular uptake and simultaneous release of both drugs, resulting in synergistic anticancer efficacy with suppressed tumor cell growth [31].

The main objective of this study was to design an oral co-delivery system for hydrophilic PMX and hydrophobic QCN based on multiple w/o/w NE, which is highly absorbable via enhanced cellular uptake due to the improved intestinal permeability of PMX and increased aqueous solubility of QCN. To enhance intestinal membrane permeability, PMX was ionically complexed with *N*^α^-deoxycholyl-l-lysyl-methylester (DCK), forming PMX/DCK. DCK has been shown to enhance the permeability of hydrophilic or macromolecular drugs by increasing the lipophilicity of drug molecules and interacting with bile acid transporters, resulting in an increase in the drug concentration gradient through the intestinal wall [32,33]. In addition, the aqueous solubility of QCN was enhanced by incorporation into the oil phase of a stable multiple w/o/w NE, which can also increase the cellular uptake of QCN. After assessing the combined effect of PMX/DCK and QCN in terms of inhibition of cancer cell proliferation and migration, the permeability of the PMX/DCK- and QCN-loaded NE system (PMX/DCK-QCN-NE) across an artificial intestinal membrane and a Caco-2 cell monolayer was evaluated. Subsequently, cellular uptake of PMX/DCK-QCN-NE into Caco-2 and apical sodium bile acid transporter (ASBT)-expressing Madin–Darby canine kidney (MDCK) cells was observed. Finally, oral bioavailability and synergistic tumor growth suppression efficacy were evaluated after oral administration of PMX/DCK-QCN-NE.

## 2. Materials and Methods

### 2.1. Materials

PMX disodium hemipentahydrate was purchased from Shipla Medicare (Karnataka, India). Caprylocaproyl macrogol-8-glycerides (Labrasol) and oleoyl polyoxylglycerides (Labrafil M 1944 CS) were provided by Gattefossé (Saint Priest, France). QCN (purity > 95%), polyoxyethylene (80) sorbitan monolaurate (Tween 80), polyethylene glycol 400 (PEG 400), deoxycholic acid (DOCA), 2-hydroxypropyl-beta-cyclodextrin (HP-beta-CD), phalloidin-tetramethylrhodamine B isothiocyanate, 4′,6-diamidino-2-phenylindole (DAPI), 2,4-diamino-*N*,10-methylpteroic acid 4-(*N*-[2,4-diamino-6-pterinidinyl-methyl]-*N*-methylamino) benzoic acid hemihydrochloride hydrate (DAMPA; internal standard [IS] for PMX), and baicalin (IS for QCN) were purchased from Sigma-Aldrich (St. Louis, MO, USA). Polyoxyethylene (160) polyoxypropylene (30) glycol (poloxamer 188; P188) and polyethoxylated castor oil (Cremophor EL) were obtained from BASF (Ludwigshafen, Germany). N-Hydroxysuccinimide ester-labeled fluorescein was obtained from Thermo Scientific (Rockford, IL, USA). All other chemicals used were of analytical grade and were used as received.

### 2.2. Animals

Sprague–Dawley rats (males, 200–250 g) and BALB/c nude mice (females, 20–25 g) were purchased from Orientbio (Gwangju, Republic of Korea). Ethical approval for this study was obtained from the Institutional Animal Care and Use Committee (IACUC) of Mokpo National University (Jeonnam, Republic of Korea). All animal experiments were performed in accordance with the National Institutes of Health Guidelines for the Care and Use of Laboratory Animals and the guidelines of the IACUC.

### 2.3. Preparation and Characterization of PMX/DCK-QCN-NE

To improve intestinal permeability, PMX was made into an ion-paired complex with DCK, which was synthesized by conjugation of cationic lysine to DOCA, as described previously [34]. Briefly, PMX solution was prepared by dissolving 50 mg PMX in 10 mL deionized water along with HP-beta-CD (163.3 mg) and P188 (50 mg), with continuous stirring; HP-beta-CD and P188 were used as dispersants for PMX/DCK in aqueous medium. Then, 10 mL of an aqueous solution of DCK (6.68 mg/mL) was added dropwise to PMX solution with continuous stirring at a 1:1 molar ratio. The mixture was then freeze dried at −70 °C for complete removal of water. After preparation of PMX/DCK, a low-energy spontaneous emulsification method was used to prepare a w/o/w NE, as described previously [35]. The optimum w/o NE was selected from the pseudo-ternary phase diagram and consisted of 19% aqueous solution of PMX/DCK, 56% surfactant (Labrasol) and co-surfactant (Tween 80) mixture (S_mix,1_; 1:1, *w*/*w*), and 25% oil phase (Labrafil M 1944 CS). Next, the aqueous phase titration method was employed to prepare a w/o/w NE containing PMX/DCK and QCN using the primary w/o NE as an oil phase. QCN (5 mg) was dispersed in 200 mg of the primary NE containing PMX/DCK equivalent to 10 mg PMX and 600 mg of a mixture of surfactant (Tween 80) and co-surfactants (Cremophor EL:PEG 400, 1.13:1, *w*/*w*) (S_mix,__2_; 1:1, *w*/*w*). Finally, 400 mg deionized water was added to the mixture as an outer aqueous phase of the w/o/w NE. The stable w/o/w NE formation was also confirmed by self-nanoemulsification efficiency test using United States Pharmacopeia dissolution apparatus II (paddle) rotating at 50 rpm. One milliliter of the w/o/w NE was gently added to 500 mL of deionized water at 37 ± 0.5 °C and visually evaluated for precipitation or phase separation.

The mean droplet size, polydispersity index (PDI), and zeta potential of the optimum formulation for PMX/DCK-QCN-NE were then measured using a Zetasizer Nano ZS (Malvern Instruments, Malvern, UK). PMX/DCK-NE or PMX/DCK-QCN-NE was diluted with deionized water (1:20), followed by vortex mixing for 1 min to minimize multiple scattering effects. The measurements of droplet size and zeta potential were performed at 25 °C with a fixed angle of 90°. In addition, the shape, surface morphology, and droplet size of the selected w/o/w NE were determined by high-resolution transmission electron microscopy (TEM). PMX/DCK-NE or PMX/DCK-QCN-NE was diluted by 100 with deionized water, and a drop of each NE was placed on a copper grid. After removing the excess with filter paper, one drop of 2% aqueous solution of phosphotungstic acid was added onto the grid to allow negative staining. The excess was removed with filter paper and the grid was observed by TEM (JEM-200; JEOL, Tokyo, Japan).

### 2.4. In Vitro Cytotoxicity

The dose-dependent effects of PMX and PMX/DCK, alone and in combination with QCN, on cancer cell viability were evaluated in human lung carcinoma (A549) cells using the WST-1 (4-[3-[4-iodophenyl]-2-[4-nitro-phenyl]-2H-5-tetrazolio]-1,3-benzene disulfonate tetrazolium salt) assay reagent. Briefly, A549 cells were seeded at a density of 5 × 10^3^ cells/well in 100 µL Roswell Park Memorial Institute medium supplemented with 10% fetal bovine serum and 1% penicillin/streptomycin in 96-well plates, and then incubated for 24 h at 37 °C in an atmosphere of 95% air and 5% CO_2_. The culture media were replaced with 100 µL PMX or PMX/DCK in free RPMI equivalent to 0.05, 0.1, and 1 µg/mL PMX or in combinations with 5, 10, and 20 µg/mL QCN in RPMI containing 0.1% (*v*/*v*) DMSO. After 72 h incubation, viable cells were evaluated using the WST-1 assay. The results for the treated cells are expressed as the percentage of viable cells compared with untreated controls.

### 2.5. In Vitro Apoptosis Assay

Apoptosis assays were also performed to determine the synergistic ability of PMX, PMX/DCK, and QCN alone and in combination to induce caspase-3/7 activity. Briefly, 5 × 10^3^ A549 cells in F12-K medium were seeded into each well of an Essen ImageLock 96-well plate (Essen Bioscience, Ann Arbor, MI, USA) and allowed to grow overnight so that the cells reached ~25–30% confluence. The cells were then treated with PMX and PMX/DCK (equivalent to 0.1 µg/mL PMX) alone and in combination with 20 µg/mL QCN in medium with caspase-3/7 green reagent at a final concentration of 5 µM, and cultured for an additional 72 h. Finally, the apoptotic effect was examined using the IncuCyte live cell analysis system (Essen Bioscience).

### 2.6. In Vitro Inhibitory Effect on Cancer Cell Proliferation and Migration

An in vitro wound healing assay was performed to investigate the synergistic inhibitory effects of PMX or PMX/DCK in combination with QCN on cancer cell proliferation and migration. To achieve this, 3 × 10^4^ A549 cells were seeded per well on a collagen-coated Essen ImageLock 96-well plate and then incubated at 37 °C for 48 h to form a confluent monolayer. A linear wound area was created in each well by careful scratching using a wound maker, and the detached cells from the monolayer were washed twice with phosphate-buffered saline (PBS; pH 7.4). The cells were further treated with PMX or PMX/DCK equivalent to 0.05, 0.1, and 1 µg/mL PMX or in combination with 5, 10, and 20 µg/mL QCN. Plates were incubated in the IncuCyte live cell analysis system at 37 °C for 60 h, and cell edges were detected every 12 h using IncuCyte Zoom image analysis software. Finally, relative wound density was monitored to quantify the synergistic efficacy of the drug combinations in impeding cancer cell proliferation and migration.

### 2.7. In Vitro Permeability Across an Artificial Intestinal Membrane and Caco-2 Cell Monolayer

The in vitro intestinal membrane permeability of PMX and PMX/DCK in water, PMX/DCK-loaded w/o/w NE (PMX/DCK-NE), QCN dispersed in water and in 0.3% NaCMC, and QCN-loaded w/o/w NE (QCN-NE) were evaluated by parallel artificial membrane permeability assay (BD Biosciences, San Jose, CA, USA) as described previously [36]. Briefly, PMX and QCN samples were diluted with PBS (pH 6.8) to final concentrations of 200 µg/mL and 400 µg/mL, respectively. Diluted samples were loaded into the wells of the donor plate (200 µL/well), and PBS (pH 6.8) was loaded into the acceptor plate (300 µL/well). The donor plate was coupled with the acceptor plate and incubated for 5 h at room temperature without agitation. After incubation, the amounts of PMX and PMX/DCK that had permeated through the artificial membrane were quantified by high-performance liquid chromatography (HPLC) with a C18 column (4.6 mm × 250 mm, 5 μm, 100 Å; 20-μL sample injection) at 25 °C. PMX and PMX/DCK were detected at 254 nm by an isocratic elution method with water (pH 3.5, adjusted with orthophosphoric acid)-acetonitrile (80:20, *v*/*v*) as the mobile phase, at a flow rate of 1 mL/min. The permeated QCN was also measured using an HPLC system with a C18 column (4.6 mm × 150 mm, 5 μm, 100 Å; 20 μL sample injection) at 35 °C. The mobile phase consisted of a mixture of water (2% acetic acid, pH 2.6) and acetonitrile at a ratio of 60:40 (*v*/*v*), which was run at a flow rate of 1 mL/min. QCN was measured using an ultraviolet (UV) detector at 370 nm. The effective permeability (*P_e_*) of the samples was calculated using the following formula:

*P_e_ =* −ln[1 − C_A_(*t*)/C_equilibrium_]/[A × (1/V_D_ + 1/V_A_) × *t*] (1)

where A is the effective filter area, V_D_ is the donor well volume, V_A_ is the acceptor well volume, *t* is the total incubation time in seconds, C_A_(*t*) is the concentration of drug in the receptor well at time *t*, and C_equilibrium_ represents [C_D_(*t*) × V_D_ + C_A_(*t*) × V_A_]/(V_D_ + V_A_), where C_D_(*t*) is the concentration of drug in the donor well at time *t*.

Similarly, the permeability values for PMX and PMX/DCK in water, PMX/DCK-NE, QCN dispersed in water and in 0.3% NaCMC, and QCN-NE across a Caco-2 cell monolayer were investigated as described previously [37]. Caco-2 cells at a density of 3 × 10^5^ cells/well were seeded onto 12-well Transwell^®^ filter inserts in complete Dulbecco’s modified Eagle medium (DMEM), and allowed to grow and differentiate for 21–29 days to form a confluent monolayer. The apical and basolateral compartments were then stabilized with 0.5 mL and 1.5 mL Hanks’ balanced salt solution (HBSS), respectively, at 37 °C for 20 min. HBSS in the apical compartment was replaced with 0.5 mL of PMX or PMX/DCK in HBSS, PMX/DCK-NE diluted with HBSS, QCN dispersed in HBSS or in HBSS containing 0.3% NaCMC, and QCN-NE diluted with HBSS (to the final concentration equivalent to 200 µg/mL PMX and 400 µg/mL QCN), and the plate assembly was incubated at 37 °C. Then, 200 µL sample was withdrawn from each basolateral compartment and replaced with 200 µL fresh HBSS at 0.5, 1, 2, 3, 4, and 5 h. The amount of PMX or PMX/DCK permeated through the Caco-2 monolayer was determined by HPLC with a UV detector, as described above. The withdrawn samples of QCN were mixed immediately with the same volume of 10 mM acetic acid to acidify and stabilize the QCN in aqueous solution, and filtered using a 0.45 µm membrane filter. Then, 20 µL baicalin in methanol (12.5 µg/mL, IS) and 2 mL ethyl acetate were added to the mixture. After vortex mixing for 2 min, the organic phase was collected in a glass vial and evaporated using a centrifugal evaporator (Genevac Ltd., Ipswich, UK). The dried residue was reconstituted in 100 µL methanol and vortexed for 4 min. The permeated QCN was then quantified by liquid chromatography/mass spectrometry (LC/MS) with a C18 column (4.6 × 150 mm, 5 µm, 100 Å) at 30°C, with acetonitrile–water (0.1% formic acid; 40:60 *v*/*v*) serving as the mobile phase, at a flow rate of 0.8 mL/min. QCN and IS ionization were performed by electrospray ionization in the negative mode with a capillary voltage ±3500 V, drying gas flow of 3.1 mL/min, and drying gas temperature of 300 °C. Protonated molecular ions were chosen to quantify QCN and baicalin at *m/z* 301.3 and *m/z* 445.4, respectively.

The apparent permeability coefficients (*P_app_*) of PMX, PMX/DCK, and QCN were calculated using the following equation:

*P_app_ =* dQ/d*t* × 1/(A × C_0_) (2)

where dQ/d*t* is the steady-state flux (μmol/s), C_0_ is the initial concentration of PMX, PMX/DCK, or QCN on the apical side (μmol/mL), and A is the surface area of the filter (cm^2^).

### 2.8. Cellular Uptake by Caco-2 and ASBT-Transfected MDCK Cells

The enhancement of cellular uptake of PMX after ion-pairing complex formation with DCK and further incorporation of PMX/DCK and QCN into the multiple NE system was evaluated in Caco-2 cells. Briefly, Caco-2 cells were seeded at a density of 5 × 10^4^ cells/coverslip and allowed to grow until formation of a monolayer. The culture media were replaced with PMX or PMX/DCK conjugated with fluorescein isothiocyanate (FITC; FITC-PMX and FITC-PMX/DCK, respectively) and their NEs (FITC-PMX-NE and FITC-PMX/DCK-NE) or QCN in 0.3% NaCMC and QCN-NE containing coumarin-6 at a concentration equivalent to 100 µg/mL PMX or QCN. After incubation at 37 °C for 0.5 h, the cells were treated with phalloidin-rhodamine (100 nM) to stain the actin filaments on the Caco-2 cells. At the end of the treatment, the cells were washed three times with PBS (pH 7.4), and cellular uptake was observed by confocal laser scanning microscopy (CLSM; Carl Zeiss, Oberkochen, Germany).

The improvement in the intestinal membrane permeability of PMX/DCK via interaction with bile acid transporter was assessed using human ASBT gene-transfected MDCK cells. MDCK cells (1 × 10^4^) were seeded onto a Cell-Tak-coated coverslip, transfected with human ASBT gene using Lipofectamine 2000^®^, and grown until the formation of a confluent monolayer. Then, the transfected cells were treated with 100 µL FITC-PMX, FITC-PMX/DCK, FITC-PMX-NE, or FITC-PMX/DCK-NE diluted with DMEM (each containing 100 µg/mL PMX) and incubated at 37 °C for 1 h. After cell fixing and blocking with 4% cold paraformaldehyde and blocking buffer (0.3% Triton X-100 and 10% normal goat serum in PBS, pH 7.4), respectively, the cells were treated with anti-human ASBT antibody. Further staining with 10 µg/mL Alexa Fluor 546-labeled secondary antibody was performed for 1 h, and nuclei were counterstained with DAPI (1 µg/mL) for 5 min. Finally, fluorescence images were obtained by CLSM.

### 2.9. Oral Absorption in Rats

The improvements in oral absorption of PMX and QCN after ionic complex formation with DCK followed by incorporation into the NE system were evaluated after oral administration of 400 µL PMX in water (50 mg/kg), PMX/DCK in water (50 mg/kg PMX), PMX/DCK-QCN-NE (50 mg/kg PMX), QCN in 0.3% NaCMC (40 mg/kg), or PMX/DCK-QCN-NE (40 mg/kg QCN) to rats. After oral administration, 150 µL of blood samples were collected at predetermined time intervals from the retro-orbital plexus and centrifuged immediately (2500 × g, 15 min, 4 °C) after mixing with 50 μL 3.8% sodium citrate solution. The isolated plasma samples were stored at −70 °C until analysis.

The concentration of PMX in plasma was measured by LC/MS, as described previously [38] with some modifications. Briefly, the defrosted plasma samples were mixed with 300 µL 2% NH_4_OH, 100 µL standard solution or plasma sample, and 10 µL DAMPA (5 µg/mL, IS). The mixture was then subjected to solid-phase extraction using a Plexa Bond Elut PAX cartridge (30 mg, 1 mL; Agilent Technologies, Santa Clara, CA, USA). After the adsorbed drug in the cartridge was eluted with 2 × 250 µL 5% formic acid in methanol, the sample was dried using a centrifugal evaporator (Genevac Ltd., Ipswich, Suffolk, UK). The dried residue was reconstituted with 100 µL 5% formic acid prepared in methanol, and the concentration of PMX in plasma was determined by LC/MS. The stationary phase consisted of a C18 column (100 mm × 2 mm, 3 µm) kept at room temperature, and the mobile phase (acetonitrile:0.34% formic acid, 15:85, *v*/*v*) was run at a flow rate of 0.2 mL/min. The drug and IS were ionized using an atmospheric pressure ionization-electron spray source in positive ion mode under the following source conditions: capillary voltage, 3.5 kV; drying gas flow rate, 3.1 L/min; and drying gas temperature, 300 °C. Quantitative analysis was performed by secondary ion MS at *m/z* 428 for PMX and *m/z* 326.1 for DAMPA.

QCN in the plasma was estimated by solvent extraction using LC/MS. The standard and plasma samples were mixed with an equal volume of 2 mol/mL hydrochloric acid, hydrolyzed in a water bath at 80 °C for 30 min, and mixed with 20 µL baicalin (IS, 1 µg/mL) along with 2 mL ethyl acetate. After mixing, the samples were centrifuged (13,000 rpm, 5 min, 4 °C), and the clear organic phase was collected and dried. The obtained residues were dissolved with 100 µL methanol and subjected to LC/MS, as described above.

### 2.10. In Vivo Antitumor Efficacy in Mice

The in vivo antitumor potentials of orally administered PMX, PMX/DCK-NE, and PMX/DCK-QCN-NE were evaluated by inducing tumors in BALB/c nude mice by subcutaneous inoculation into the flanks of A549 cells at a concentration of 1 × 10^6^ cells/100 μL PBS (pH 7.4). Once the tumor mass reached 80–100 mm^3^, mice were divided randomly into four groups (10 mice per group): control (untreated), PMX (once-a-day oral administration of 20 mg/kg PMX), PMX/DCK-NE (once-a-day oral administration based on 20 mg/kg PMX), and PMX/DCK-QCN-NE (once-a-day oral administration based on 20 mg/kg PMX and 10 mg/kg QCN). Tumor volumes and body weights in all four groups were measured every 3 days. Tumor volume was calculated as a^2^ × b × 0.52, where a is the width and b is the length. Four weeks after administration, mice were sacrificed and the tumor masses were isolated for histological examination. To examine microvessel expression, tumor sections were immunostained using an antibody to the epithelial cell marker CD31, which showed brown staining of endothelial cells or their clusters. In addition, in vivo tumor cell apoptosis and proliferating cells in the tumor sections were examined by staining of the sections for proliferating cell nuclear antigen and by terminal deoxynucleotidyl transferase-mediated dUPT nick end labeling (TUNEL), respectively.

### 2.11. Pharmacokinetic and Statistical Analyses

Pharmacokinetic parameters were determined using a non-compartmental method with WinNonlin^®^ software (version 5.3; Certara Inc., Princeton, NJ, USA). All data were evaluated using the *t* test for comparisons between two mean values for unpaired data or one-way analysis of variance followed by Tukey’s multiple-comparisons test among three or more mean values for unpaired data, and expressed as mean ± standard deviation. In all analyses, *p* < 0.05 was taken to indicate statistical significance.

## 3. Results and Discussion

### 3.1. Preparation and Characterization of PMX/DCK-QCN-NE

To improve the intestinal membrane permeability and oral bioavailability of PMX, an ion-pairing complex of PMX with DCK was formed and then incorporated into the inner aqueous phase of a w/o/w multiple NE. QCN was also loaded into the oil phase of an NE to improve its aqueous solubility and permeability. To obtain the optimum NE formulation for co-delivery of PMX/DCK and QCN, different surfactants, co-surfactants, and oils were screened to determine their solubility and self-emulsification ability to form a single-phase NE with reduced interfacial energy and no precipitation. Labrasol and Tween 80, Cremophor EL and PEG 400, and Labrafil M 1944 CS were selected as surfactants, co-surfactants, and oil phase, respectively, for construction of pseudo-ternary phase diagrams (data not shown). Based on the ability of the inner phase to disperse the maximum amount of hydrophilic drug, a surfactant to co-surfactant weight ratio for the primary NE (S_mix,1_) of 1:1 was selected (Labrasol:Tween 80) (Supplementary Materials Figure S1A). Furthermore, S_mix,2_, with a weight ratio of surfactant (Tween 80) to co-surfactant mixture (Cremophor EL + PEG 400) of 1:4, exhibited the maximum self-nanoemulsifying region as well as potential to be diluted infinitely with the GI fluid without phase separation due to enhanced penetration of the oil phase into surfactant monomers, thus increasing the fluidity of the interface (Supplementary Materials Figure S1B). In addition, the selection of S_mix_ depends on the necessary extent of minimizing the oil-water interfacial tension and increasing the dispersion entropy of the system [39]. The concentrations of PMX/DCK and QCN in NE were determined based on the maximum drug loading capacity with minimum concentration of S_mix_ that produced transparent NE on infinite dilution as well as possessed high artificial intestinal membrane permeability. Finally, self-nanoemulsification test confirmed the thermodynamic stability of the w/o/w NE with no sign of phase separation and precipitation upon infinite dilution with deionized water.

**Figure 1:**
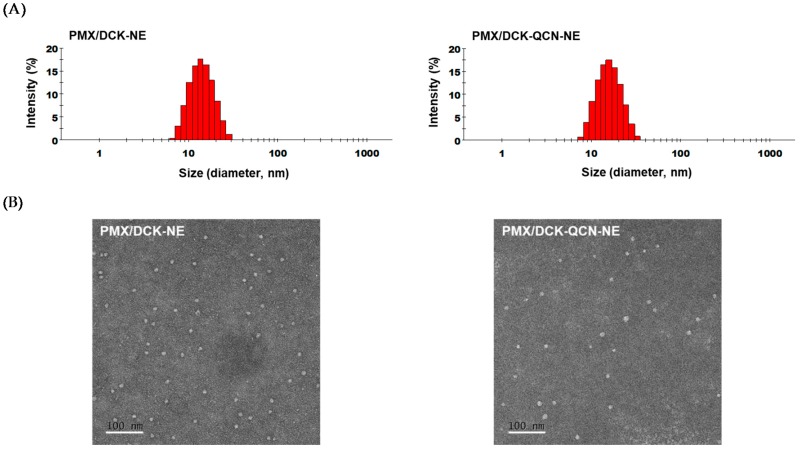
Droplet size distribution (**A**) and transmission electron micrographs (**B**) of pemetrexed/*N*^α^-deoxycholyl-l-lysyl-methylester-loaded nanoemulsion (PMX/DCK-NE) and pemetrexed/*N*^α^-deoxycholyl-l-lysyl-methylester- and quercetin-loaded nanoemulsion (PMX/DCK-QCN-NE). Scale bar represents 100 nm.

The optimum NE containing only PMX/DCK had a droplet size, PDI, and zeta potential of 13.2 ± 0.132 nm, 0.095 ± 0.015, and −3.99 ± 1.11 mV, respectively (Figure 1A). Incorporation of QCN into the oil phase of the NE did not alter the droplet size, PDI, or zeta potential of the formulation (14.6 ± 0.205 nm, 0.085 ± 0.010, and −4.63 ± 0.028 mV, respectively), indicating that QCN was distributed completely in the oil phase. The narrow size distribution might be due to the composition of NE selected from the metastable region in the pseudo-ternary phase diagram, resulting in forming nano-sized droplets and having resistance to physical destabilization, such as phase separation and precipitation [40]. In addition, droplet size of w/o/w NE prepared by low-energy spontaneous emulsification can be controlled by varying the concentrations of components and lowering the interfacial tension. The morphology and surface structure of PMX/DCK-NE and PMX/DCK-QCN-NE, as determined by TEM, also suggested the formation of homogenous nano-sized droplets with diameters <50 nm, in accordance with the findings obtained with a particle size analyzer (Figure 1B). These results confirmed that S_mix_ induced thermodynamic stability of the NE by reducing the interfacial energy between the immiscible liquids [41]. Moreover, after 3 months of storage of PMX/DCK-QCN-NE at 25 ± 2 °C (60 ± 5% relative humidity), the drug content in the NE was maintained at >97%; there were no precipitation or phase separation, and change in either droplet size or the PDI (data not shown).

### 3.2. In Vitro Cytotoxic Effects of PMX, PMX/DCK, and QCN

**Figure 2:**
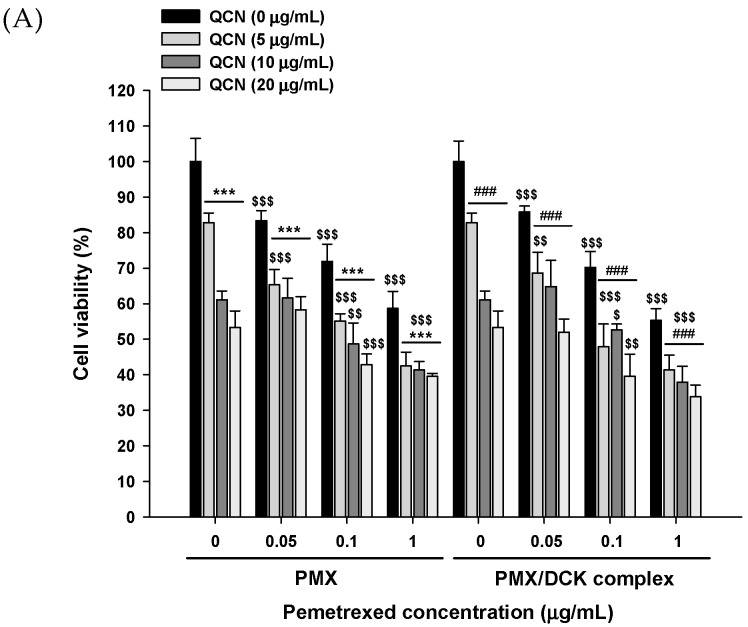

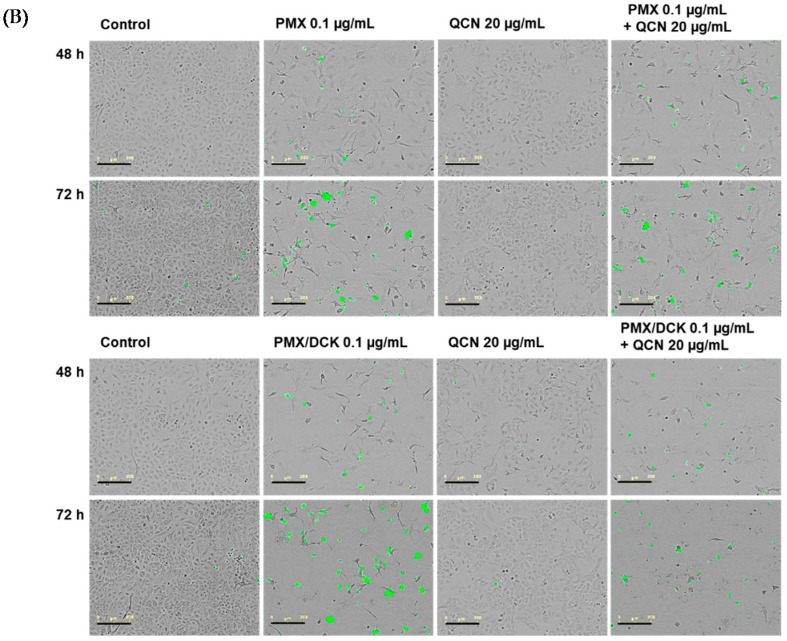
(**A**) In vitro cytotoxic effects of PMX, PMX/DCK, QCN, and combinations of PMX or PMX/DCK with QCN on A549 cells after incubation for 48 h (*n* = 5). *** *p* < 0.001 compared with PMX alone at the same concentration. ^###^ *p* < 0.001 compared with PMX/DCK alone at the same concentration equivalent of PMX. ^$^ *p* < 0.05, ^$$^ *p* < 0.01, ^$$$^ *p* < 0.001 compared with QCN alone at the same concentration; (**B**) Live cell fluorescence images of caspase-3/7 activity at 48 and 72 h after treatment of A549 cells with PMX, PMX/DCK, QCN, and combinations of PMX or PMX/DCK with QCN. Scale bar represents 200 μm.

Cell viability assays were performed using A549 cells to determine the synergistic cytotoxic effects of different concentrations of PMX or PMX/DCK with QCN. PMX and PMX/DCK exhibited dose-dependent reductions in cell viability, which suggested no change in the cytotoxic effect of PMX after complex formation with DCK (Figure 2A). PMX and PMX/DCK at 1 µg/mL PMX showed significant reductions of cell viability of 58.7% ± 4.76% and 55.3% ± 3.25%, respectively. Exposure of A549 cells to QCN also showed dose-dependent cytotoxicity, with cell viability values of 61.0% ± 2.51% and 53.4% ± 4.62% after treatment with 10 µg/mL and 20 µg/mL QCN, respectively, indicating that PMX or PMX/DCK with QCN effectively inhibited A549 cell growth as a single agent. In addition, cells treated simultaneously with PMX (0.1 µg/mL) and QCN (20 µg/mL) showed a significant reduction in viability (42.8% ± 3.02%), which was 1.67- and 1.25-fold lower than those of PMX and QCN, respectively. Similarly, the cell viability with PMX/DCK (equivalent to 0.1 µg/mL PMX) was 70.2% ± 4.48%, representing a decrease of 43.6% after cells were treated with 20 µg/mL QCN (39.6% ± 6.17%). These results indicated synergistic cytotoxic effects of PMX or PMX/DCK in combination with QCN, with different anticancer activity. PMX is known to inhibit folate-dependent enzymes, resulting in the disruption of nucleotide synthesis. On the other hand, dose-dependent cytotoxic activity of QCN was shown to be induced by cell-cycle arrest at the G2/M and G1/S transitions, suppression of cyclin-dependent kinases, disassembly of the interphase microtubule cytoskeleton, and inhibition of cell survival pathways [4].

We further examined the apoptotic efficacies of PMX or PMX/DCK in combination with QCN in A549 cells. Cells treated with PMX or PMX/DCK showed considerable decreases in the number of viable cells at 48 h, with further reduction in viable cell numbers at 72 h after treatment, compared with the control and QCN treatment groups (Figure 2B). PMX/DCK-treated cells showed slightly higher caspase-3/7 activity than did those treated with PMX, which may have been related to the increase in lipophilicity of PMX by DCK, resulting in enhanced cellular uptake. In addition, combined treatment of cells with PMX or PMX/DCK and QCN induced significant apoptotic activity, and most A549 cells had died at 72 h after treatment. The role of QCN in the induction of apoptosis has been studied in many cancer cell lines [42,43]. QCN was shown to inhibit metabolic activity and to decrease the level of p53 expression, with simultaneous reductions in anti-apoptotic protein expression [44]. However, cells treated with QCN (20 µg/mL) showed no significant change in caspase-3/7 activity up to 72 h, suggesting the existence of molecular mechanisms of apoptosis other than caspase-3/7. It was reported that QCN-induced apoptosis in A549 cells was accompanied by a decrease in the B cell lymphoma 2 (BCL2)/BCL2-associated X protein ratio and changes in the tubulin, actin, and vimentin cytoskeletons [4]. Therefore, further studies are required to determine the precise mechanism of QCN-induced apoptosis.

### 3.3. In Vitro Inhibition of Cancer Cell Proliferation and Migration

**Figure 3:**
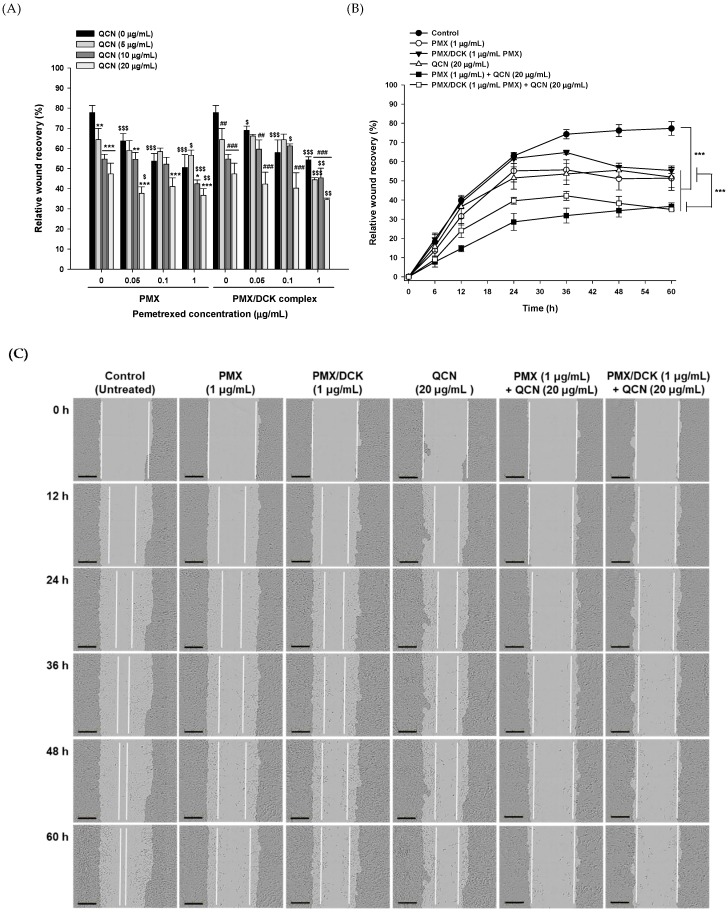
In vitro effects of PMX, PMX/DCK, QCN, and combinations of PMX or PMX/DCK with QCN on the proliferation/migration of A549 cells. (**A**) Wound closure after 48 h treatment (*n* = 5; * *p* < 0.05, ** *p* < 0.01, *** *p* < 0.001 compared with PMX alone at the same concentration; ^##^ *p* < 0.01, ^###^ *p* < 0.001 compared with PMX/DCK alone at the same concentration equivalent of PMX; ^$^ *p* < 0.05, ^$$^ *p* < 0.01, ^$$$^ *p* < 0.001 compared with QCN alone at the same concentration); (**B**) Time course of closure of the wounded areas after treatment of the cells with PMX, PMX/DCK, QCN, and combinations of PMX or PMX/DCK with QCN (*n* = 5; *** *p* < 0.001); (**C**) Representative images taken at different time points after treatment of the cells with PMX, PMX/DCK, QCN, and combinations of PMX or PMX/DCK with QCN. White lines represent the cell-proliferation/migration progress. Scale bar represents 300 μm.

Next, we examined the synergistic inhibitory activities of PMX or PMX/DCK in combination with QCN in cancer cell proliferation/migration using a wound healing assay. PMX and PMX/DCK showed dose- and time-dependent inhibition of relative wound recovery, whereas cells with no drug treatment showed significant increases in cell proliferation/migration-driven wound closure up to 60 h. Treatment with PMX or PMX/DCK (equivalent to 1 µg/mL PMX) for 60 h significantly reduced relative wound closure, with values of 50.6% ± 6.38% and 54.3% ± 1.48%, respectively, which were lower than those for PMX and PMX/DCK at 0.1 µg/mL. Similarly, QCN also showed dose-dependent inhibition of A549 cell proliferation/migration (47.4% ± 5.31%) at 20 µg/mL, which was 13.4% lower than that after treatment with QCN at 10 µg/mL. In addition, combined treatment with PMX (0.1 µg/mL) and QCN (20 µg/mL) resulted in relative wound recovery of 41.0% ± 4.31%, which was lower than that seen with the same concentrations of PMX (53.7% ± 3.82%) and QCN (47.3% ± 5.31%). Furthermore, the relative wound recovery of cells treated with PMX/DCK (0.1 µg/mL) and QCN (20 µg/mL) was 40.3% ± 7.64%, which represented higher sensitivity than seen with PMX/DCK and QCN, with reductions in cell proliferation/migration of 30.5% and 14.9%, respectively (Figure 3A). Increasing the concentration of PMX/DCK to 1 µg/mL and simultaneous treatment with 20 µg/mL QCN also resulted in significant dose-dependent inhibition of relative wound recovery of 34.6% ± 0.71% at 60 h. This effect may have been due to the synergistic effects of PMX and QCN in inhibiting cell proliferation. PMX inhibits biosynthesis of purines and pyrimidines, thereby inducing an imbalance in the nucleotide pool and resulting in DNA damage. Moreover, it upregulates expression of the tumor suppressor genes, p21 and p53, and increases apoptosis and the c-caspase 3 protein level. QCN induces cell-cycle arrest along with DNA fragmentation, both resulting in delayed wound closure [45,46,47]. Wound recovery was significantly delayed and cancer cell proliferation/migration was significantly impeded at 24 h after treatment in all treatment groups compared with the control group (Figure 3B). The scratched monolayers treated with PMX and PMX/DCK (equivalent to 1 µg/mL PMX) showed relative wound densities of 55.7% ± 5.29% and 64.8% ± 1.15%, respectively, at 36 h after treatment. In the QCN (20 µg/mL) treatment group, 53.6% ± 5.55% of the cell free zone had recovered at 36 h, but only 31.9% ± 3.89% and 42.2% ± 2.34% of the scratched areas were covered at 36 h after treatment of the cells with 1 µg/mL PMX and PMX/DCK, respectively, in combination with QCN (20 µg/mL). Thus, treatment with PMX and PMX/DCK, alone and in combination with QCN, resulted in the presence of sizeable gaps in the cell monolayer up to 60 h, representing significant suppression of wound recovery compared with controls (Figure 3C).

### 3.4. In Vitro Membrane Permeability

The intestinal membrane permeability of PMX after conjugation with DCK was significantly increased by 2.53-fold compared with free PMX. Further, incorporation of PMX/DCK into NE resulted in 116% and 448% greater permeability compared with PMX/DCK and PMX, respectively (Table 1). QCN-NE showed maximal permeation through the artificial membrane, with permeability values 16.6- and 13.8-fold greater than those of QCN in water and in 0.3% NaCMC, respectively (Table 1).

The permeability of PMX across a Caco-2 cell monolayer after preparation of PMX as an ion-paring complex with DCK (PMX/DCK) was also significantly improved by 7.58-fold compared with free PMX. Moreover, the permeability of PMX/DCK-NE was 1.33- and 10.1-fold greater than those of PMX/DCK and PMX, respectively (Table 1). In addition, incorporation of QCN into the NE also resulted in 1438% and 283% higher *P_app_* compared with QCN in water and in 0.3% NaCMC, respectively (Table 1). The enhanced permeability of PMX may be due to the increased lipophilicity of PMX caused by DCK, which improved partitioning into the cell membrane. The enhanced permeability of QCN may be attributable to increased solubility and surface area of the drug caused by the presence of surfactant and co-surfactant mixture, which enhanced contact with biological membranes and disrupted cell membrane integrity, thereby increasing membrane fluidity [19]. Furthermore, the use of Labrasol and Cremophor EL in formulations was shown to increase the intestinal membrane permeability of drug molecules through opening or loosening of epithelial tight junctions via interaction with zonula occludens-1 and filamentous actin [48,49,50].

### 3.5. Uptake into Caco-2 and ASBT-Expressing MDCK Cells

**Figure 4:**
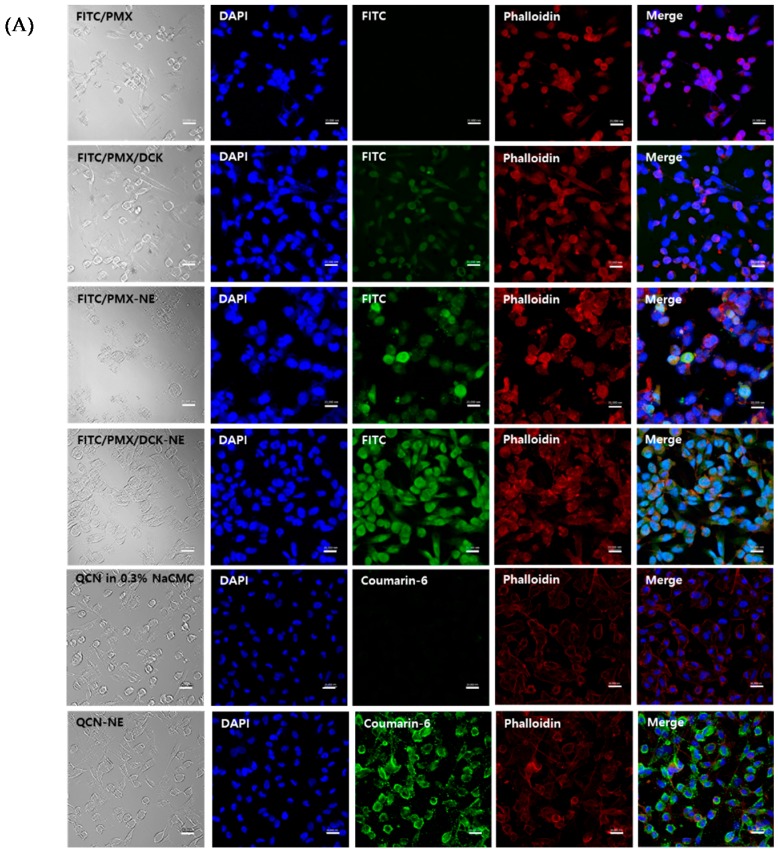

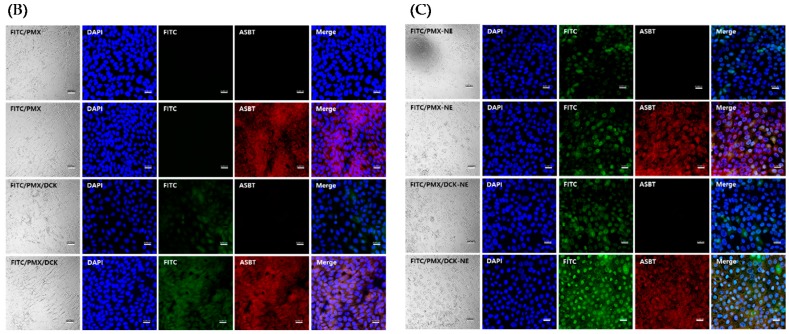
Confocal laser scanning microscopy images of the cellular uptake of (**A**) fluorescein isothiocyanate (FITC)-PMX, FITC-PMX/DCK, FITC-PMX-NE, FITC-PMX/DCK-NE, QCN in 0.3% NaCMC, and QCN-NE in Caco-2 cells; (**B**) FITC-PMX and FITC-PMX/DCK in MDCK or ASBT-transfected MDCK cells; and (**C**) FITC-PMX-NE and FITC-PMX/DCK-NE in MDCK or ASBT-transfected MDCK cells. Scale bar represents 20 µm.

Complex formation of FITC-PMX with DCK and incorporation of FITC-PMX/DCK into the NE system resulted in significantly greater cellular uptake into Caco-2 cells compared with that observed with FITC-PMX (Figure 4A). This result was attributed to the increased lipophilicity of PMX by the permeation enhancer (DCK), which was also shown to be able to alter the membrane structure and fluidity, resulting in enhanced cellular uptake [51]. Similarly, the intracellular uptake of QCN-NE was also significantly increased compared with that of QCN in 0.3% NaCMC (Figure 4A). These observations suggest that nano-sized oil droplets enhanced the passive transport of QCN through the intestinal walls and that formulation components, such as Labrasol and Cremophor EL, modified the properties of the epithelial barrier, thereby enhancing cellular permeability [48,52].

Intracellular uptake of FITC-PMX/DCK was more prominent in ASBT-expressing MDCK cells than in normal controls, whereas neither normal nor ASBT gene–transfected cells showed intracellular uptake of FITC-PMX (Figure 4B). This result was supported by the observation that DCK can improve the intestinal membrane permeability of poorly absorbed drugs by enhancing interaction with bile acid transporters in the ileum, which increases facilitated transport of the complexed drug molecules [53,54]. In addition, FITC-PMX-NE exhibited more cellular uptake in ASBT-expressing and normal MDCK cells than did FITC-PMX and FITC-PMX/DCK, which may have been due to the effects of formulation components in altering cell membrane permeability. However, incorporation of the ion-paired FITC-PMX/DCK into the NE resulted in the most prominent intracellular uptake into ASBT-expressing MDCK cells compared with normal MDCK cells, as well as into transfected and non-transfected cells treated with FITC-PMX-NE (Figure 4C). Therefore, the maximum cellular uptake observed with FITC-PMX/DCK-NE may have been due to the synergistic action of DCK, which can be transported through bile acid transporters, and NE formulation components (Labrasol, Cremophor EL, and Tween 80), which alter the cell membrane structure and fluidity.

### 3.6. Oral Absorption in Rats

**Figure 5:**
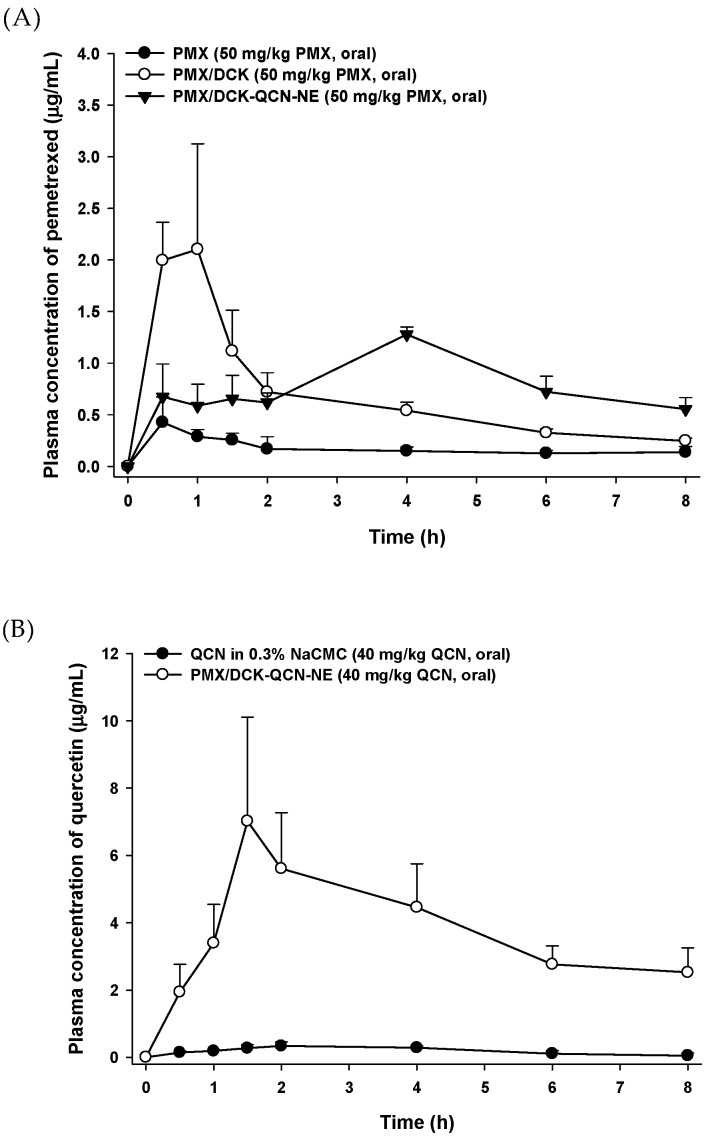
The mean plasma concentration-time profiles of PMX and QCN after single oral administration of (**A**) PMX (50 mg/kg), PMX/DCK (equivalent to 50 mg/kg PMX), or PMX/DCK PMX/DCK-QCN-NE (equivalent to 50 mg/kg PMX) and (**B**) QCN in 0.3% NaCMC (40 mg/kg) or PMX/DCK-QCN-NE (equivalent to 50 mg/kg PMX) to rats. Values are expressed as mean ± standard deviation (*n* = 4).

The pharmacokinetic parameters of PMX and QCN after single oral administration of PMX, PMX/DCK, QCN in 0.3% NaCMC, and PMX/DCK-QCN-NE in rats are summarized in Table 2. Compared with oral PMX alone, the maximum plasma concentration (C_max_) and area under the plasma concentration-time curve (AUC) achieved after oral administration of PMX/DCK (equivalent to 50 mg/kg PMX) were 2.28 ± 1.05 µg/mL and 5.49 ± 1.01 µg·h/mL, respectively, representing increases of 404% and 295%, respectively. The increased AUC may have been due to the enhanced lipophilic properties and bile acid transporter-mediated penetration of PMX by ionically complexed DCK molecules, leading to the increased intestinal absorption of PMX. In addition, DOCA in DCK has been shown to disrupt the intestinal barrier by compromising dephosphorylation and by cytoskeletal rearrangement at the epithelial lining-tight junction level, thereby increasing paracellular transport of the drug [55,56]. Moreover, after oral administration of PMX/DCK-QCN-NE, the AUC_last_ of PMX was increased by 1.15-fold compared with that of PMX/DCK, resulting in 4.51-fold greater oral bioavailability of PMX compared with PMX alone. These observations confirmed the synergistic effects of DCK and NE components in increasing the intestinal permeability of PMX (Figure 5A). However, a slight decrease of 0.44-fold in the C_max_ value was observed after incorporation of PMX/DCK into the NE. Furthermore, the time to reach the maximum concentration was increased by 6.00-fold for PMX/DCK-QCN-NE compared with oral PMX/DCK, suggesting delayed absorption of PMX after the encapsulation of PMX/DCK into the NE.

The C_max_ and AUC_last_ values for QCN after oral administration of PMX/DCK-QCN-NE were also increased by 22.0- and 23.9-fold, respectively, compared with oral QCN dispersed in 0.3% NaCMC, indicating a significant increase in intestinal absorption of QCN after incorporation into the NE (Figure 5B). The increment in absorptive transport of QCN may be attributable to enhanced aqueous solubility and intestinal permeability of QCN caused by the effects of the surfactant mixture on the integrity of tight junctions and intestinal membrane structure via interaction with filamentous actin and zonula occludens-1 without significant intestinal membrane damage [57], and to the NE-induced increase in surface area for drug absorption and enhancement of lymphatic transport [58].

### 3.7. In Vivo Tumor Growth Inhibition Effect

**Figure 6:**
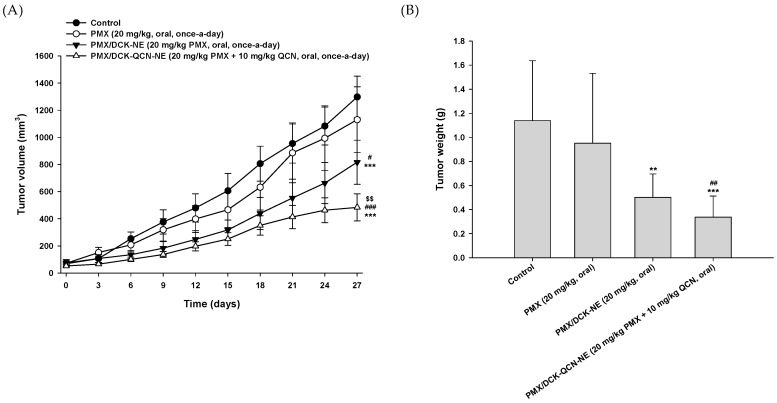

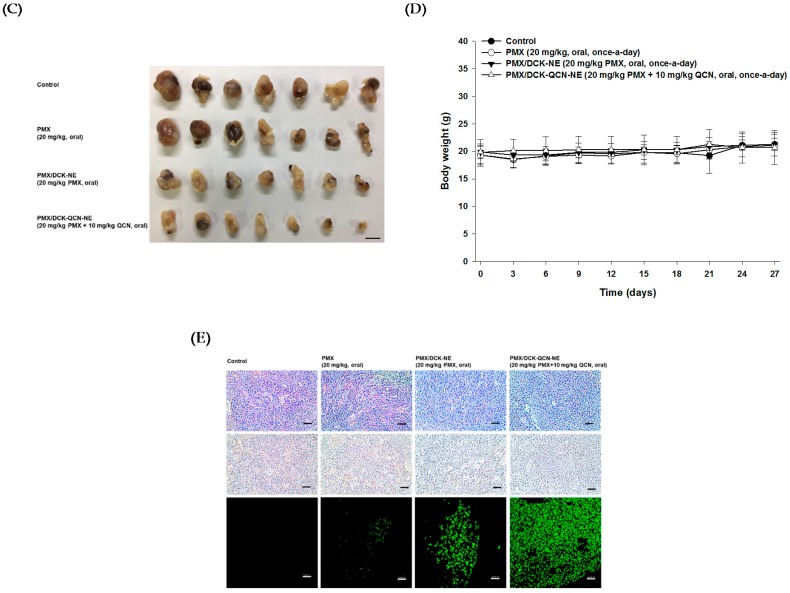
In vivo tumor growth inhibition efficacy in A549 tumor-bearing mice after treatment with once-daily oral administration of PMX (20 mg/kg), PMX/DCK-NE (equivalent to 20 mg/kg PMX), or PMX/DCK-QCN-NE (equivalent to 20 mg/kg PMX and 10 mg/kg QCN) for 27 days. (**A**) Tumor volume in mice (*n* = 10; *** *p* < 0.001 compared with the control group; ^#^ *p* < 0.05, ^###^ *p* < 0.001 compared with the oral PMX group; ^$$^ *p* < 0.01 compared with the oral PMX/DCK-NE group); (**B**) Variation in body weight in mice during treatment (*n* = 10); (**C**) Isolated tumor weight in A549 tumor-bearing mice (*n* = 10; ** *p* < 0.01, *** *p* < 0.001 compared with the control group; ^##^ *p* < 0.01 compared with the oral PMX group); (**D**) Photographs of isolated tumors from each group on day 21. Scale bar represents 10 mm; (**E**) Representative cross-sectional images of isolated tumor tissues stained with anti-CD31 antibody for microvessels (brown), PCNA for proliferating cells (brown), and TUNEL for apoptosis (green fluorescence) at 27 days after treatment. Scale bar represents 50 µm.

The synergistic tumor growth inhibitory effect of co-delivery of PMX and QCN was also confirmed after oral administration of PMX/DCK-QCN-NE to mice bearing A549 cells. In a preliminary study, we observed tumor suppression efficacy of the NE comprising PMX/DCK or QCN alone at a dose of >20 mg/kg PMX or >10 mg/kg QCN (data not shown). Therefore, we compared the anti-cancer effect in A549 cell-bearing mice after once-daily oral administration of PMX/DCK-QCN-NE (equivalent to 20 mg/kg PMX and 10 mg/kg QCN) to those after once-daily oral administration of 20 mg/kg PMX or PMX/DCK-NE (equivalent to 20 mg/kg PMX). The tumor volume was not decreased in mice treated with oral PMX alone in comparison with the control group (Figure 6A). On the other hand, after treatment with oral PMX/DCK-NE for 27 days, the tumor volume was significantly reduced by 1.38- and 1.59-fold compared with the oral PMX and control groups, respectively, indicating enhanced oral absorption of PMX by complex formation with DCK, as well as inclusion of PMX/DCK in the NE. In addition, tumor growth was inhibited more significantly after incorporation of QCN into the PMX/DCK-NE, which resulted in maximal reduction of tumor volume by 40.7% and 62.7% compared with the oral PMX/DCK-NE and control groups, respectively. The oral administration of PMX/DCK-QCN-NE also significantly decreased isolated tumor mass, with a weight on day 27 that was 56% and 70% lower than those in the oral PMX/DCK-NE and control groups, respectively (Figure 6B,C). However, no significant change in body weight was observed in any treatment group, suggesting that the administered drugs and formulations had no systemic toxicity (Figure 6D).

Histological evaluation of isolated tumor tissues stained with anti-CD31 antibody showed a similar tendency, with a marked decrease in CD31-positive microvessels in the PMX/DCK-NE treatment group compared with the oral PMX group. Moreover, incorporation of QCN into the PMX/DCK-NE resulted in significantly greater anti-angiogenesis efficacy than that of PMX/DCK-NE and PMX. In addition, the number of proliferating cells was markedly decreased after treatment with PMX/DCK-NE, and no proliferating cells were observed in tissues from the PMX/DCK-QCN-NE-treated group. The TUNEL assay results also showed similar trends, with significantly greater numbers of apoptotic cells in PMX/DCK-QCN-NE compared with PMX and PMX/DCK-NE (Figure 6E). However, further studies are required to identify an optimum dosing regimen for synergistic tumor growth inhibition efficacy of PMX/DCK-QCN-NE in various cancer cell-bearing models.

To our knowledge, this report is the first to describe a synergistic inhibitory effect of PMX and QCN in combination on cancer cell proliferation/migration, as well as enhancement of intestinal membrane permeability and oral bioavailability of both drugs by formulation into a w/o/w multiple NE. Based on the findings presented here, incorporation of QCN into the PMX/DCK-NE resulted in significant inhibitory activity on tumor growth; this formulation may therefore be suitable as a drug carrier for co-delivery of hydrophilic and hydrophobic drugs via the oral route.

## 4. Conclusions

In this study, a w/o/w multiple NE was developed for co-delivery of PMX and QCN, and was shown to improve their oral absorption along with having synergistic anticancer activities. PMX and PMX/DCK in combination with QCN showed concentration-dependent synergistic inhibitory effects on cancer cell proliferation/migration. The artificial intestinal membrane and Caco-2 cell permeability of PMX was improved after ion-pairing complex formation with the permeation enhancer, DCK. The levels of oral absorption of PMX/DCK and QCN were more significantly enhanced by incorporation into the multiple NE, which may have been due to increased lipophilicity and facilitated transport of PMX via interaction of DCK with bile acid transporters, as well as changes in the intestinal membrane structure and fluidity induced by surfactants and co-surfactants in the NE. Therefore, the oral bioavailability of PMX and QCN in rats was increased by 4.51- and 23.9-fold compared with that of free PMX and QCN, respectively, with the result that orally administered PMX/DCK-QCN-NE maximally suppressed tumor growth in A549 cell-bearing mice by 62.7% compared with the control group. These observations suggest that PMX/DCK-QCN-NE is a promising nanocarrier for co-delivery of PMX and QCN via the oral route to achieve synergistic antitumor efficacy as well as long-term chemotherapy.

## Figures and Tables

**Table 1:** Effective permeability and apparent permeability of PMX, PMX/DCK-NE, QCN, and QCN-NE.

Test Material	Effective Permeability Across an Artificial Membrane (*P_e_*, ×10^−6^, cm/s)	Apparent Permeability Across a Caco-2 Cell Monolayer (*P_app_*, ×10^−6^, cm/s)
PMX in water	6.03 ± 1.41	1.57 ± 0.749
PMX/DCK	15.3 ± 3.65 ***	11.9 ± 1.47 ***
PMX/DCK-NE	33.1 ± 0.739 ***^,###^	15.8 ± 3.53 ***^,#^
QCN in water	0.000± 0.000	0.550 ± 0.032
QCN in 0.3% NaCMC	1.20 ± 0.527 **	2.21 ± 0.533
QCN-NE	16.6 ± 0.621 ***^,$$$^	8.46 ± 2.21 ***^,$$$^

Values are expressed as mean ± standard deviation (*n* = 6). ** *p* < 0.01, *** *p* < 0.001 compared with PMX or QCN in water; ^#^ *p* < 0.05, ^###^ *p* < 0.001 compared with PMX/DCK; ^$$$^ *p* < 0.001 compared with QCN in 0.3% NaCMC.

**Table 2:** Pharmacokinetic parameters of PMX and QCN in rats after oral administration of PMX, QCN in 0.3% NaCMC, or PMX/DCK-QCN-NE.

Test Material	PMX			QCN	
	PMX in Water	PMX/DCK in Water	PMX/DCK-QCN-NE	QCN in 0.3% NaCMC	PMX/DCK-QCN-NE
Administration	Oral	Oral	Oral	Oral	Oral
Dose of PMX or QCN (mg/kg)	50	50	50	40	40
*T*_max_ (h)	0.833 ± 0.577	0.667 ± 0.289	4.00 ± 0.000 ***^,###^	2.00 ± 0.000	1.67 ± 0.289
*T*_1/2_ (h)	6.06 ± 1.83	4.10 ± 1.40	3.34 ± 0.589	4.83 ± 2.68	4.76 ± 0.551
*C*_max_ (μg/mL)	0.452 ± 0.221	2.28 ± 1.05 **	1.28 ± 0.072	0.336 ± 0.122	7.38 ± 3.03 ^$$^
AUC_last_ (μg∙h/mL)	1.39 ± 0.395	5.49 ± 1.01 ***	6.29 ± 0.677 ***	1.26 ± 0.665	30.1 ± 7.57 ^$$$^
AUC_inf_ (μg∙h/mL)	2.56 ± 0.755	6.99 ± 1.63 **	9.02 ± 1.38 ***	2.60 ± 1.03	47.6 ± 12.9 ^$$$^
Relative bioavailability	1.00	3.94 ± 0.727 ***	4.51 ± 0.486 ***	1.00	23.9 ± 6.00 ^$$$^

*T*_max_, time to reach maximum plasma concentration; *T*_1/2_, half-life of plasma concentration; *C*_max_, maximum plasma concentration; AUC_last_, area under the plasma concentration-time curve from zero to the time of the last measurable plasma concentration; AUC_inf_, area under the plasma concentration-time curve from zero to infinity; Relative bioavailability, (AUC_last, PMX in test material_/Dose_PMX in test material_)/(AUC_last, PMX in water_/Dose_PMX in water_) or (AUC_last, QCN in test material_/Dose_QCN in test material_)/(AUC_last, QCN in 0.3% NaCMC_/Dose_QCN in 0.3% NaCMC_). Values ar expressed as mean ± standard deviation (*n* = 4). ** *p* < 0.01, *** *p* < 0.001 compared with PMX in water; ^###^ *p* < 0.001 compared with PMX/DCK; ^$$$^ *p* < 0.001, ^$$$^ *p* < 0.001 compared with QCN in 0.3% NaCMC.

## Supplementary Materials

The following are available online at http://www.mdpi.com/1999-4923/10/3/158/s1, Figure S1: (A) Pseudo-ternary phase diagrams of water-in-oil (w/o) nanoemulsion (primary nanoemulsion) region using Labrafil M 1944 CS (oil phase), water (aqueous phase), Labrasol (surfactant), and Tween 80 (co-surfactant) with a different mixture of surfactant and co-surfactant (Smix,1) ratio of (a) Smix,1 2:1, (b) Smix,1 1:1, and (c) Smix,1 1:2. (B) Pseudo-ternary phase diagrams of water-in-oil-in-water (w/o/w) nanoemulsion region using primary nanoemulsion (oil phase), Tween 80 (surfactant), a mixture of Cremophor EL and PEG 400 (1.13:1, *w*/*w*; co-surfactants), and water with a different mixture of surfactant and co-surfactants (Smix,2) ratio of (a) Smix,2 1:1, (b) Smix,2 1:2, (c) Smix,2 1:3, and (d) Smix,2 1:4.
