# Supplementary material for: Preparation, Characterization, and In Vivo Evaluation of an Oral Multiple Nanoemulsive System for Co-Delivery of Pemetrexed and Quercetin
Source: Pharmaceutics. 2018 Sep 12;10(3):158. doi: 10.3390/pharmaceutics10030158 (PMC6161295; doi:10.3390/pharmaceutics10030158)
Supplement: Supplementary file 1 [file pharmaceutics-10-00158-s001.pdf]

Supplemental Material

(A)

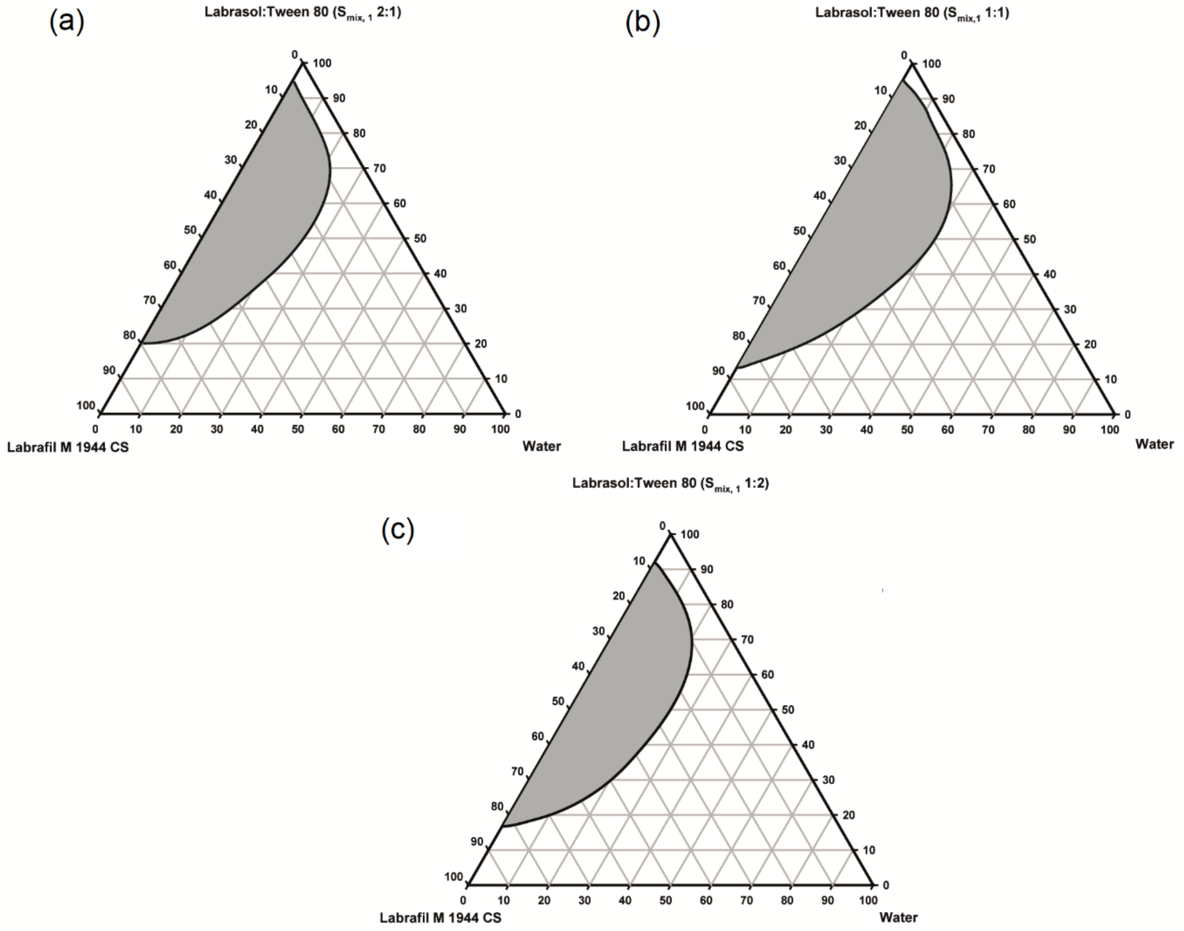

(B)

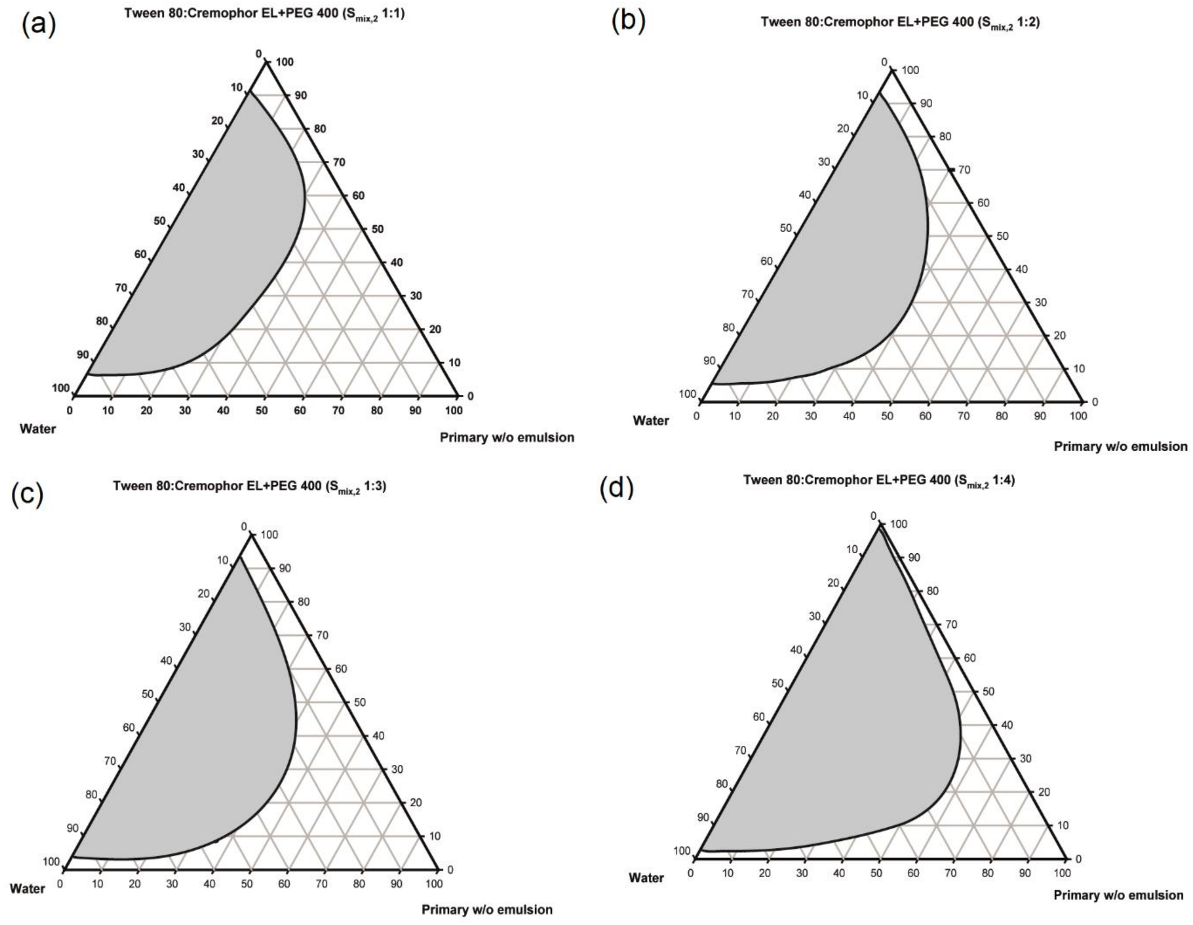

Figure S1. (**A**) Pseudo-ternary phase diagrams of water-in-oil (w/o) nanoemulsion (primary nanoemulsion) region using Labrafil M 1944 CS (oil phase), water (aqueous phase), Labrasol (surfactant), and Tween 80 (co-surfactant) with a different mixture of surfactant and co-surfactant ($S_{mix,1}$) ratio of (a) $S_{mix,1}$ 2:1, (b) $S_{mix,1}$ 1:1, and (c) $S_{mix,1}$ 1:2. (**B**) Pseudo-ternary phase diagrams of water-in-oil-in-water (w/o/w) nanoemulsion region using primary nanoemulsion (oil phase), Tween 80 (surfactant), a mixture of Cremophor EL and PEG 400 (1.13:1, w/w; co-surfactants), and water with a different mixture of surfactant and co-surfactants ($S_{mix,2}$) ratio of (a) $S_{mix,2}$ 1:1, (b) $S_{mix,2}$ 1:2, (c) $S_{mix,2}$ 1:3, and (d) $S_{mix,2}$ 1:4.
